# Apoptotic efficacy of multifaceted biosynthesized silver nanoparticles on human adenocarcinoma cells

**DOI:** 10.1038/s41598-018-32480-5

**Published:** 2018-09-25

**Authors:** Blassan Plackal Adimuriyil George, Neeraj Kumar, Heidi Abrahamse, Suprakas Sinha Ray

**Affiliations:** 1 Laser Research Centre, Faculty of Health Sciences, University of Johannesburg, Doornfontein, 2028 South Africa; 2 Department of Applied Chemistry, University of Johannesburg, Doornfontein, 2028 South Africa; 3 DST/CSIR National Centre for Nanostructured Materials, Council for Scientific and Industrial Research, Pretoria, 0001 South Africa

## Abstract

Metallic nanoparticles (NPs) especially silver (Ag) NPs have shown immense potential in medical applications due to their distinctive physio-chemical and biological properties. This article reports the conjugation of Ag NPs with *Rubus fairholmianus* extract. The modification of Ag NPs was confirmed using various physico-chemical characterization techniques. The cytotoxic effect of *Rubus*-conjugated Ag NPs (RAgNPs) was studied by LDH assay and proliferation by ATP assay. The apoptotic inducing ability of the NPs were investigated by Annexin V/PI staining, caspase 3/7 analysis, cytochrome c release, intracellular ROS analysis, Hoechst staining and mitochondrial membrane potential analysis using flow cytometry. The expression of apoptotic proteins caspase 3, Bax and P53 were analyzed using ELISA and caspase 3, Bax using western blotting. Cells treated with 10 µg/mL RAgNPs showed an increased number of cell death by microscopic analysis compared to untreated control cells. The RAgNPs induced a statistically significant dose-dependent decrease in proliferation (*p* < 0.001 for 5 and 10 µg/mL) and increased cytotoxicity in MCF-7 cells. A 1.83 fold increase in cytotoxicity was observed in cells treated with 10 µg/mL (*p* < 0.05) compared to the untreated cells. Nuclear damage and intracellular ROS production were observed upon treatment with all tested concentrations of RAgNPs and the highest concentrations (5 and 10 µg/mL) showed significant (*p* < 0.05, *p* < 0.01) expression of caspase 3, Bax and P53 proteins. The data strongly suggest that RAgNPs induces cell death in MCF-7 cells through the mitochondrial-mediated intrinsic apoptosis pathway. The present investigation supports the potential of RAgNPs in anticancer drug development.

## Introduction

Cancer is one of the most deadly diseases with high mortality rates. It refers to a collection of diseases where certain cells the body begin to grow and multiply disorderly. Nanotechnology has shown significant promise in the development and delivery of drugs that can potentially contribute to overcome many limitations of current drug^1^. Nanomedicine is the use of nanotechnology in medicine and the use of nanoparticles (NPs) for the treatment of various diseases is one of the characteristics of nanomedicine. Recent increases in the number of different nanomaterials produced is contributing to the advancement of nanomedicine in diverse fields. Nanoparticles coated or conjugated with multiple functionalities are able to target the tumor site thereby allowing the early detection and the eradication of tumors. Many noble metal and metal oxides/sulphides NPs have been studied for oncological applications including anticancer activity, nanocarriers and cellular imaging^1–5^. Among the noble metals, silver (Ag) and its complexes have attracted significant attention due to its remarkable medicinal value and display enormous efficiency as an anticancer agent^5,6^. Ag NPs are one of the promising nanoproducts commonly used in nanomedicine because of their unique properties^7,8^. Ag NPs have showed antimicrobial properties against various bacteria, fungi, protozoa and viruses^9^. Recently, the anticancer effect of AgNPs has been studied against various cancer cells^10^. Ag NPs are usually smaller than 100 nm and consist of around 20–15,000 Ag atoms^11^. Moreover, Ag nanostructures can be produced as nanorods, nanotubes, nanowires, multifacets or films. At the nano-scale, the Ag particles exhibit different physico-chemical and biological properties compared to the regular metal^12^. Additionally, multifaceted and branched Ag NPs has garnered immense research attention in various fields but they are comparatively difficult to synthesize because of isotropic cubic lattices of Ag^13^.

A number of approaches are employed for the synthesis of NPs; among them, biological methods are less toxic and eco-friendly. In the synthesis of NPs, organic molecules are used as the capping agent to aid stabilization of the NPs^14^. Among the various biosynthetic methods, the use of plant extract is desirable as they are easily accessible and contain various metabolites, which help in the reduction of silver ions, and improves the rate of synthesis. The need for NPs biosynthesis has increased due to the high cost associated with physical and chemical synthesis procedures. Moreover, the chemi-synthetic process leads to the presence of toxic chemicals on the surfaces, which have harmful effects in medical applications^15^. This problem can be overcome by green synthesis of NPs^16^. For this reason researchers are using plant extracts and phytochemicals for low-cost synthesis of NPs. With their radical scavenging or reducing properties, they are generally responsible for the reduction of metal compounds into their respective NPs. The potential of plants as biological constituents for the NPs synthesis is yet to be explored.

Considering the various limitations as well as adverse effects of current anticancer drugs, there is an urgent need to develop new classes of therapeutic agents with improved biocompatibility and efficacy. *Rubus fairholmianus*, an ethnomedicinally important tropical plant from Western Ghats of India, has demonstrated excellent cytotoxicity towards cancer cells in our previous study^17^. Cytotoxic characteristics can be attributed due to presence of organic species such as phenolics and flavonoids present in the extract^17^. Earlier studies showed that the cytotoxicity of synthesized Ag NPs is associated to the involvement of the level of cellular reactive oxygen species (ROS) and mitochondrial membrane disruption^18,19^. The size of Ag NPs synthesized using the antioxidant constituents from blackberry, blueberry, pomegranate, and turmeric extracts was found to be between 20–500 nm size, depending on the nature of extracts and method of preparation used^20^. However, there is a lack of detailed research results regarding the anticancer effects of green synthesized Ag NPs. It will be of interest to determine the synergistic cytotoxic effect of Ag NPs and *Rubus* extract on cancer cells as they could demonstrate better biocompatibility and enhanced therapeutic attributes. To the authors knowledge no study has been undertaken to determine the *in vitro* anti-proliferative effects of Ag NPs capped with *Rubus* extracts against MCF-7 breast carcinoma cells. Therefore, the current study was performed to modify Ag NPs with *Rubus* extract, and evaluate its anticancer potential against MCF-7 cells *in vitro*. The cytotoxicity, proliferation and caspase mediated apoptotic effects of capped Ag NPs were monitored through various *in vitro* analyses.

## Results and Discussion

### Structural characterisation of *Rubus* extract conjugated Ag NPs

**Figure 1:**
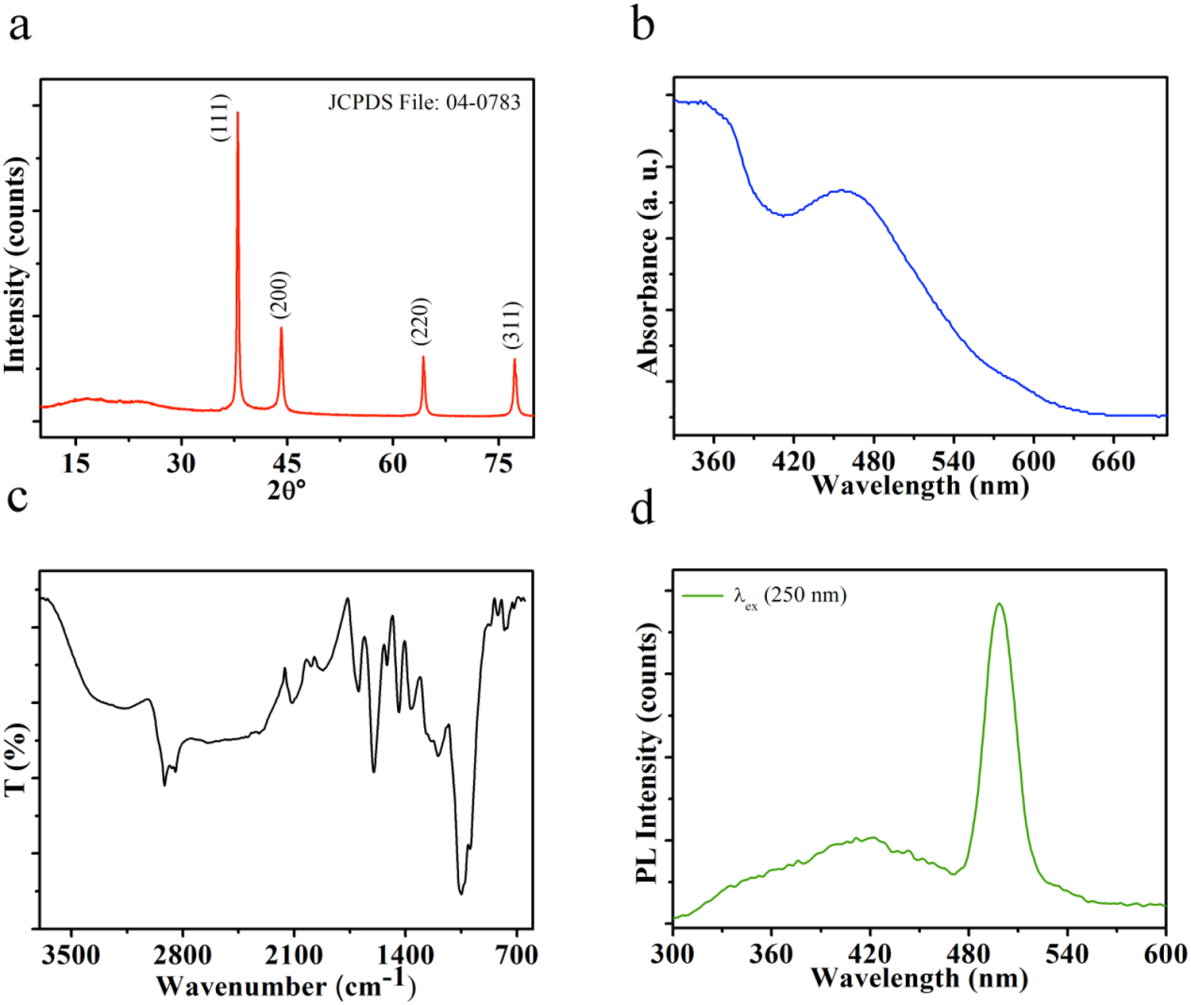
The XRD pattern (**a**), UV-vis spectrum (**b**), FTIR spectrum (**c**) and photoluminescence (PL) emission spectrum of RAgNPs (**d**).

The crystal structure and purity of as-prepared sample were examined by powder X-ray diffraction (XRD). All peaks in the diffraction pattern (Fig. 1a) were well matched with face-centred cubic phase of metallic Ag (JCPDS 04-0783)^21,22^. The prominent peaks noticed in pattern at 2θ = 38.02° (highest intensity), 44.12°, 64.33°, and 77.31° related to the (222), (200), (220) and (311) planes of Ag, respectively. The observed diffraction peaks are sharp, which shows the highly crystalline behaviour of prepared samples. The amorphous region from 12° to 30° belongs to the RFRA extracts as it contains various organic moieties and also indicates the crystallisation of bioorganic phase exist on the surface of Ag NPs.

UV-vis spectroscopy measurement (Fig. 1b) was done to investigate the reduction of metal salts into metal NPs in presence of RFRA extracts. The colour change from yellow to brown was observed due to the reduction of Ag ions to Ag NPs by active molecules of extracts. This may be attributed to the surface plasmon resonance (SPR) of as-prepared Ag NPs. The absorption spectrum revealed a maxima peak at 455 nm (λ_SPR_), confirmed the formation of Ag NPs. Generally, the previously synthesised Ag NPs demonstrated the SPR band in the region of 395–420 nm^22,23^. The red shifting of the SPR band was noticed due to *in-situ* conjugation of extract with Ag NPs to form biohybrid^24^. Broad absorption was noticed from 415 to 660 nm due to the localised SPR. It can be described by the well-known Mie resonance condition^25^. The lower wavelength absorption can be ascribed to bioorganic molecules, which are present in the RFRA extracts.

In order to determine the possible functional groups present in phytoconstituents of RFRA extracts, FTIR spectroscopy measurements were carried out. These functional groups play a vital role as reducing agents for metal salts as well as stabilisation agents for Ag NPs. The IR spectrum of RAgNPs is shown in Fig. 1c. The broad absorption band appears in the range of 3176–3358 cm^−1^ and is due to O-H and N-H stretching vibration of phytoconstituents (such as polyphenols and amides) of extracts. The peaks appearing at 2924, 2852 cm^−1^ indicate the presence of asymmetric and symmetric C-H groups, respectively. The weak absorption region form 2560–2680 cm^−1^ is arising from thiol (S-H) stretching. The characteristics peaks at 1701 and 1599 cm^−1^ corresponds to carbonyl (C=O) and amide I (N-H) and/or C=C groups, respectively. The bands of amide II and III are found at 1511 and 1366 cm^−1^, respectively. The peaks position at 1437 cm^−1^ can be ascribed to alkanes C-H bending or COO^−^ of carboxylate group. The peaks appearing in the region 1200–995 cm^−1^ are due to overlapping of C-O, C-N, C-O-C and C-O-P stretching modes. Furthermore, the absorption bands that appear below 1000 cm^−1^ are possibly attributed to sp^2^ C-H bending of alkene and aromatic regions of phytoconstituents. Thus, FTIR shows the possibility of flavanones, protein, amino acids, polyphenols, and cellulose molecules in the RFRA extracts, which are responsible for bio-reduction and stability to Ag NPs^23,26^. Coating of RFRA extracts enhances biological characteristics as well as biocompatibility with stealth nature as evident from *in vitro* cytotoxicity behaviour.

Figure 1d demonstrates the room temperature photoluminescence (PL) emission spectrum of RAgNPs with excitation wavelength (λ_ex_) of 250 nm. PL emission peak positions of Ag NPs were noticed previously over a range from 320 to 540 nm^27,28^. A well-defined strong peak was observed in the PL spectra at 498 nm for Ag NPs. The high photoluminescent intensity probably obtained due to enhancement of electron density by coating of phytoconstituents on Ag NPs. The electron density plays a major role in photoluminescence emission^28^. The excitation minimum was observed around at 467 nm, which is close to the SPR obtained in UV-vis spectroscopy measurements (Fig. 1b). It showed that the observed PL is mainly acquired from the single-electron excitations between discrete Ag energy levels rather than the SPR. The luminescence regions from 468 to 300 nm can possibly be attributed to ligand-metal charge transfer (LMCT) and plasmon mediated energy transfer between Ag NPs and phytoconstituents of extracts^29^.

**Figure 2:**
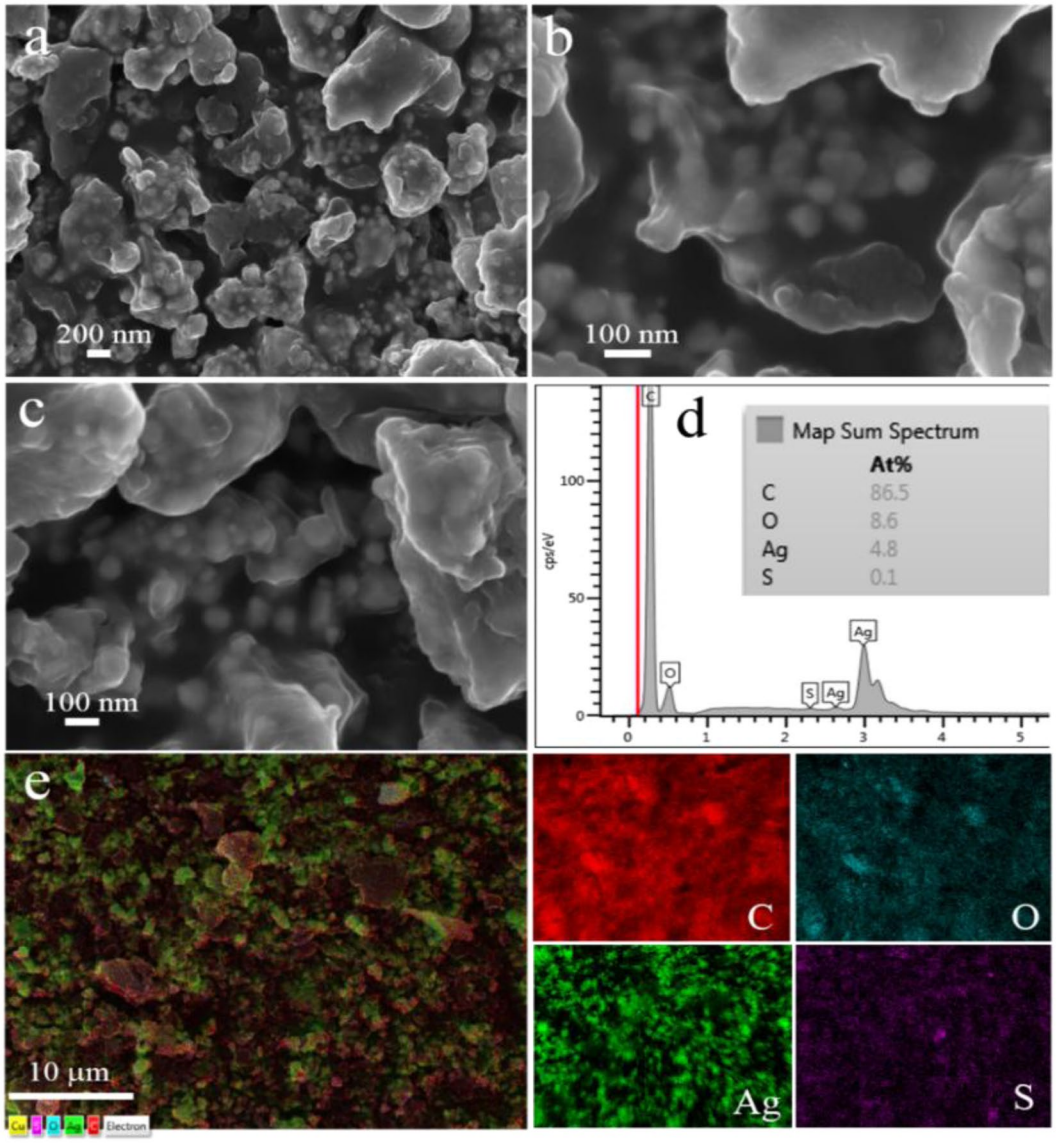
(**a**–**c**) SEM images of RAgNPs, (**d**) represents the EDX map spectrum and (**e**) overlapped EDX map followed by separate map images.

Morphology of as-prepared Ag NPs was investigated by SEM and TEM. As shown in parts (a) to (c) of Fig. 2, RAgNPs have shown random shapes such as nanorods, spherical, ellipsoidal, etc. It is noticeable that these NPs are completely covered by *Rubus* extract. The size of as-prepared NPs was less than 200 nm. To check out the composition of RAgNPs, EDX study was carried out and result showed the presence of C, Ag, O, and S elements (Fig. 2d). Ag NPs are uniformly distributed throughout the sample (as shown in EDX map images Fig. 2e). Extra elements like C, O, and S were appeared due to the presence of residues of the extract on the surface of NPs.

**Figure 3:**
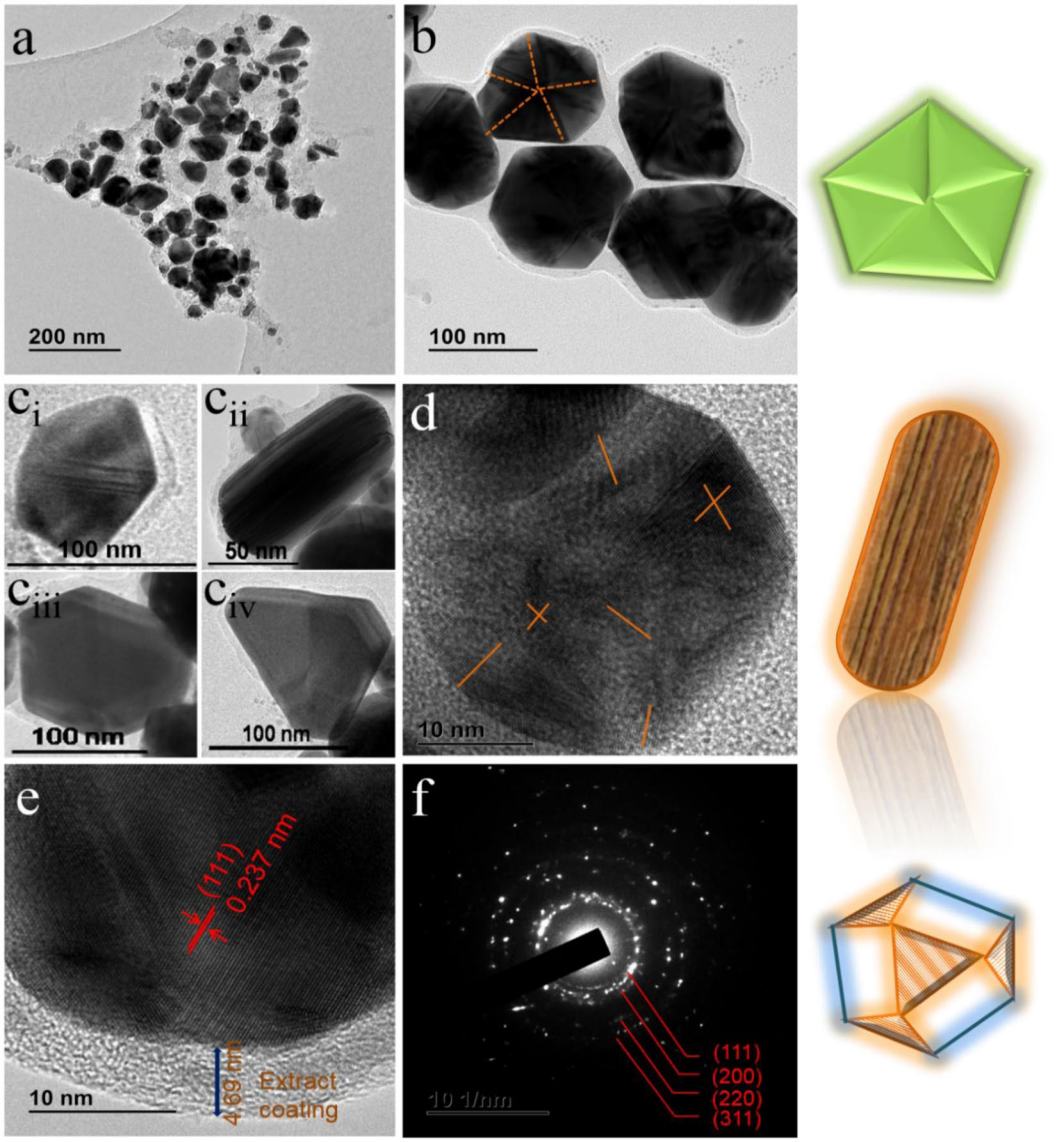
TEM images of RAgNPs: (**a**) various morphologies of Ag NPs, (**b**) truncated decahedral particles (C_i_) twinned particle, (C_ii_) nanorods, (C_iii_) cuboctahedron, (C_iv_) truncated triangular nanoplates, (**d**) HRTEM of icosahedral particle with fringes orientation directions, (**e**) HRTEM of nanorods and represent also coating surfaces (**f**) SAED image of RAgNPs. Right side shows the artistic representation of morphologies *viz*. truncated decahedral particles, nanorods and cuboctahedron (from top to down).

**Figure 4:**
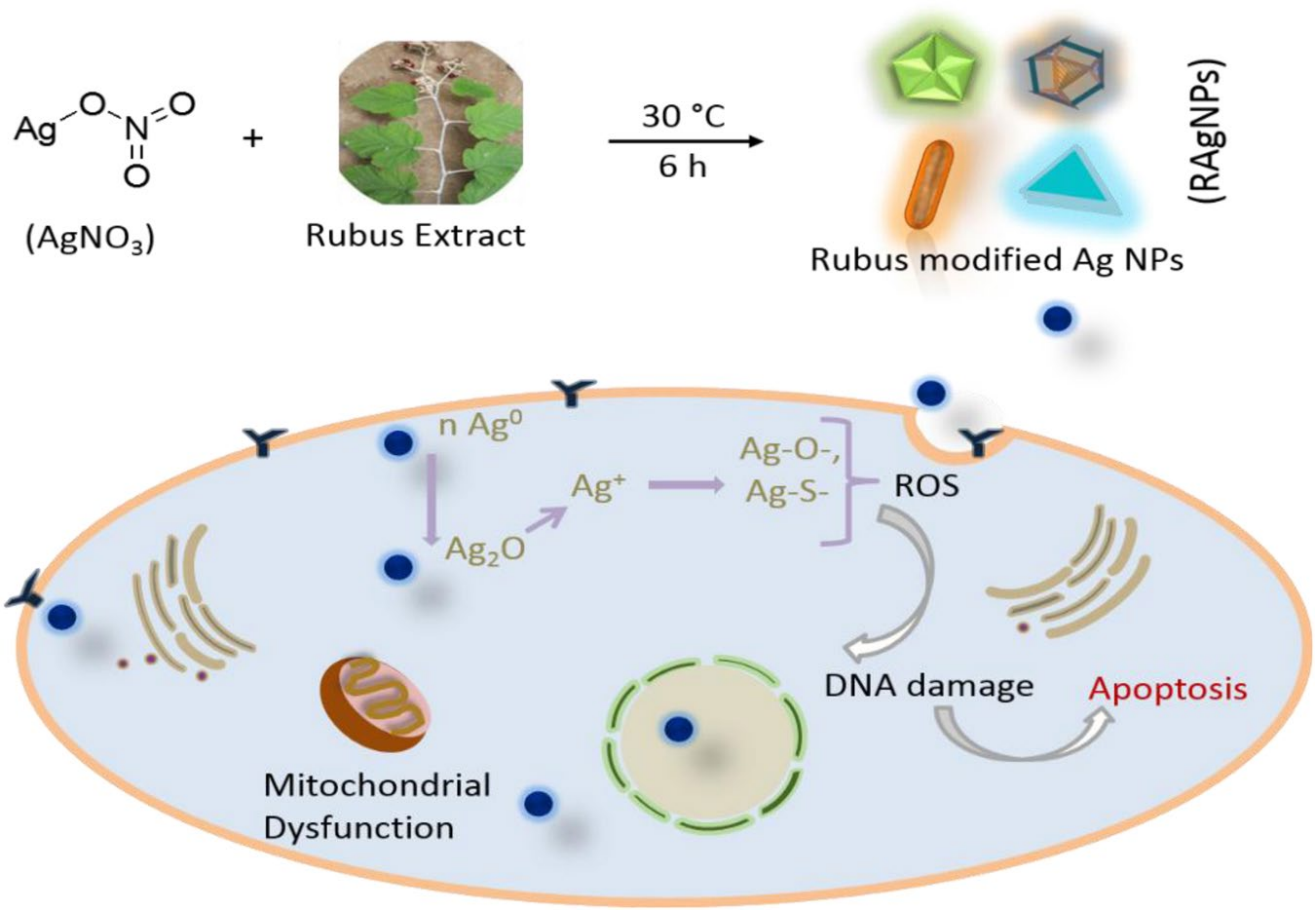
Representative reaction for RAgNPs formation and schematic for nanoparticle induced cancer cell apoptosis.

Furthermore, to investigate the detailed morphological features of prepared RAgNPs, TEM study was conducted and images are shown in Fig. 3a–f. Similar to SEM results, the surface of Ag NPs is covered by organic layer (*Rubus* extract), which further confirm the modification of NPs (Fig. 3a). Various morphologies of RAgNPs were observed including truncated decahedral particles, twinned particles, nanorods, cuboctahedron, truncated triangular nanoplates (Fig. 3b,c). The size of RAgNPs varied from ~30–150 nm with different shapes from spherical to nanorods. The appearance of various shapes and morphologies can be attributed to capping and stabilising behaviour of different functional groups such as carbonyl acids (-COOH), carboxylate (-COOR), amines (-NH_2_), amides (-CONH_2_), mercapto (-SH) and hydroxyls (-OH) of the *Rubus* extract^4,15,17,30,31^. Similar Ag NP morphologies were reported in previous literature^32,33^. HRTEM of selected RAgNPs were examined and images are shown in Fig. 3d,e. Figure 3d showed the icosahedral particle with different lattice fringes that are orientated in different directions, confirm the multifaceted characteristics of as-prepared Ag NPs. In Fig. 3e, it can clearly be seen the lattice fringes at the edge of Ag nanorods, the crystal lattice space was 0.237 nm, which belong to the (111) plane of cubic Ag. It can be seen that all NPs have been effectively coated with *Rubus* extract (with approximately 4.7 nm thickness). Representative reaction of RAgNPs formation has shown in Fig. 4. Moreover, SAED pattern of RAgNPs demonstrated the polycrystalline behaviour of as-prepared sample with clear visible spots corresponding to the main reflection lattice planes of (111), (200), (220), and (311), as similar to cubic Ag XRD pattern (Fig. 3f).

Zeta (ζ) potential of as-prepared RAgNPs dispersion in water was found to be −24.5 mV at room temperature (Fig. S1). It indicated that the dispersion has excellent stability at neutral conditions. It also showed that as-prepared NPs were stabilised by electrostatic repulsion and steric hindrance of organic moieties of *Rubus* extract.

### Morphology, proliferation and cytotoxicity

**Figure 5:**
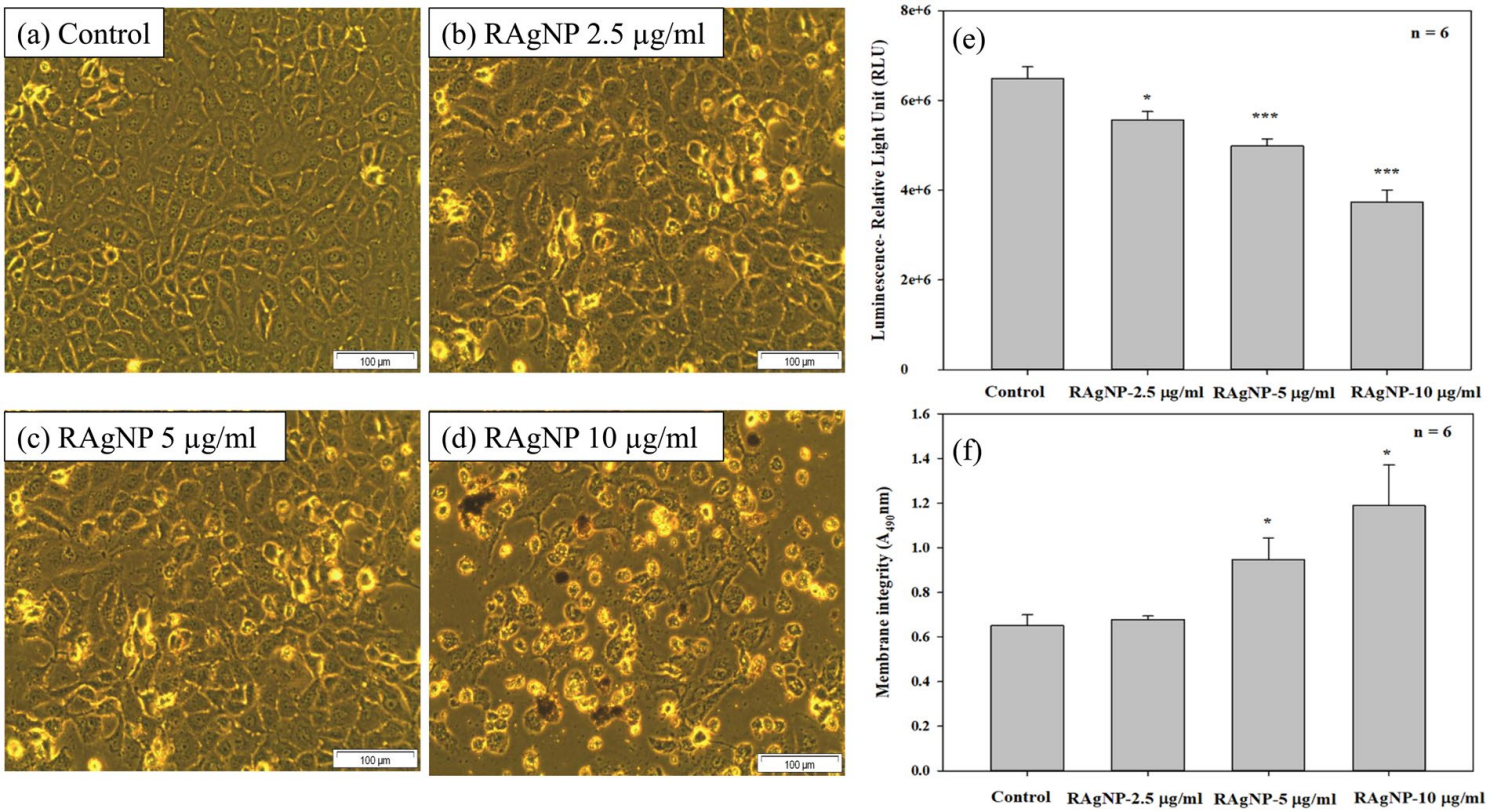
Morphological changes in MCF-7 cells after RAgNPs treatment. There were no significant visible differences in control (**a**) and 2.5 µg/mL treated groups (**b**), the cells did not show any cellular shrinkage and apoptotic bodies after the treatment. However, more dead cells were observed at higher concentrations (5 and 10 µg/mL). The treated cells showed loss of intact membrane, loss of contact with neighbouring cells, condensed, detached from the culture plate showed the features of apoptotic cells. The ATP luminescent cell proliferation assay (**e**) was used to determine MCF-7 cell proliferation after the treatment with RAgNPs. Control cells showed an increased ATP level, whereas a dose dependent significant (***p < 0.001 and **p < 0.05) decrease in ATP level was observed in experimental groups. The Lactate Dehydrogenase (LDH) cytotoxicity test (**f**) showed a significant increase in cytotoxicity of cells after the 24 h treatment with RAgNPs compared to control cells. The significant differences between treated and controls groups are shown as *p < 0.05.

Morphological variations in cells following the 24 h RAgNPs treatment were compared with the control cells (Fig. 5). The pre-treated cells presented with an irregular shape and the numbers of dead cells in the treated groups were found to be increased. Total loss of membrane integrity and detachment from the culture plate were observed in the cells treated with RAgNPs. Compared to 2.5 and 5 µg/mL treated cells 10 µg/mL treated cells were more toxic showing a higher incidence of dead cells. The most identifiable morphological features of apoptosis were observed in RAgNPs treated MCF-7 cells.

Uncontrolled cell division is a primary contributor to the progression of cancer. The energy level in MCF-7 cells remained higher, which was notable from the increased ATP level in untreated cells. Treatment of MCF-7 cells with RAgNPs caused a decrease in the intracellular ATP, which shows decreased proliferation rates. We confirmed that the proliferation of MCF-7 cells was inhibited in a concentration-dependent manner after exposure to RAgNPs (2.5, 5 and 10 µg/mL) (Fig. 5e). RAgNPs at 5 and 10 µg/mL showed significant (*p* < 0.001) antiproliferative activities on MCF-7 cell compared to 2.5 µg/mL (*p* < 0.05). However, all the doses were significant in reducing the cellular proliferation compared with control cells.

Similar effects were also found when the LDH assay was performed to check the cytotoxic effects of RAgNPs on MCF-7 cells. The LDH assay was used to measure the cellular membrane integrity following treatment with RAgNPs. The MCF-7 cell membrane damage was measured by the release of LDH to the culture medium. The untreated cells displayed low levels of LDH release related to the treated cells. A significant (*p* < 0.05) increase in toxicity was noticed at higher concentrations (5 and 10 µg/mL) of RAgNPs (Fig. 5f). A 1.83 fold increase in cytotoxicity was observed in MCF-7 cells when treated with 10 µg/mL compared to the 5 and 2.5 µg/mL of RAgNPs (1.44 and 1.04). Thus, our results suggest that RAgNPs were able to decrease the breast cancer cells while increasing the cytotoxicity after 24 h treatment.

AgNPs synthesised from various plant extracts have shown to induce cytotoxicity in MCF-7 cells and possess anticancer activities. Morphological features such as loss of membrane integrity, cytoplasmic condensation, and cell clumping, were observed in MCF-7 cells treated with AgNPs^18,19,34,35^.

### Apoptotic analysis

**Figure 6:**
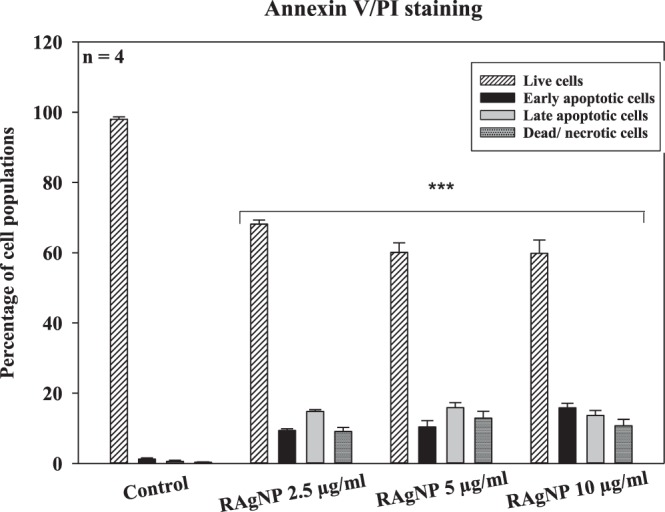
Annexin V FITC/PI staining was used to assess the mode of cell death. RAgNPs treated MCF-7 cells showed an increased percentage of apoptotic population after 24 h incubation. The population of early and late apoptotic cells percentage in the control group found to be lower and the live cells percentage was higher in control cells compared with experimental groups. A significant decrease (***p < 0.001) in the live cells and increase in the early, late apoptosis and dead cells percentage were observed in treated MCF-7 cells.

Apoptosis plays a vital role in the homeostasis and several morphological and biochemical changes in cells characterize this process. The RAgNPs statistically significantly (*p* < 0.001) induced cell death in a dose-dependent manner after 24 h treatment, the Annexin V/PI staining determined the population of apoptotic and non-apoptotic population of cells. The results of staining (Fig. 6) displayed a substantial increased uptake of Annexin V by an increased cell population percentage in the lower and upper right quadrants in the pre-treated groups. RAgNPs significantly (*p* < 0.001) induced early cell apoptosis (Annexin V +/PI−) (9.35 ± 0.52, 10.38 ± 1.83 and 15.83 ± 1.29%) and late apoptosis (Annexin V +/PI+) (14.78 ± 0.53, 15.88 ± 1.41 and 13.65 ± 1.44%) in MCF-7 cells at 2.5, 5 and 10 µg/mL concentrations, indicative of apoptotic cell death. In contrast, the early, late apoptotic and necrotic/dead cell percentages (Annexin V−/PI+) (1.23 ± 0.33, 0.60 ± 0.29 and 0.33 ± 0.09) in untreated cells were found to be very low compared to RAgNPs treatments, while the percentages of viable cells (Annexin V−/PI−) decreased upon the treatment with NPs.

**Figure 7:**
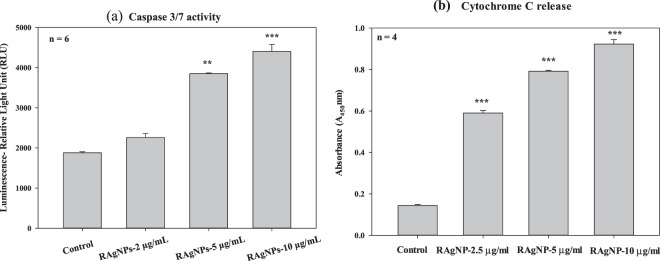
Caspase 3/7 activity (**a**) was determined as a function of caspase dependent apoptosis in cells after the 24 h treatment. There was a highly significant (*p* < 0.001) increase in caspase 3/7 activity after 10 µg/mL RAgNPs treatments compared to 2.5 and 5 µg/mLconcentrations and control cells. Cytochrome c release (**b**) is an important measure of cellular damage. RAgNPs treated cells showed significant (***p < 0.001) release of cytochrome c compared to control cells. The significant differences between treated and controls groups are shown as ***p < 0.001.

Upregulation of caspases, apoptotic mediators, were observed after exposure of MCF-7 to the RAgNPs. The activation of caspases (3 and 7) were measured in cells treated with three different doses of RAgNPs after 24 h incubation. The levels of caspase expression were compared with the untreated cells arbitrarily set to 1.0. The results revealed that RAgNPs increased caspase 3/7 upregulation at various doses while 10 µg/mL showed the highest caspase 3/7 activity (Fig. 7a). At 10 μg/mL of RAgNPs, caspase 3/7 activity extended to a maximum of 1.18 fold increase after 24 h incubation (*p* < 0.001). Similar results were observed at 5 μg/mL (1.09 fold increase) (*p* < 0.01). Even though a similar trend was observed in all concentrations, higher dose showed a significant (*p* < 0.001) increase in caspase 3/7 activity.

Cytochrome c release is an important event in the execution phase of cell damage via the intrinsic apoptotic pathway. A difference in various proapoptotic and apoptotic proteins in cells lead to damage of the mitochondrial membrane, resulting in discharge of cytochrome c and followed by caspase 3 activation. The release of cytochrome c was determined after 24 h RAgNPs treatment. The ELISA results of cytochrome c release revealed that in the control, the cells were unable to initiate such a damaging event and low cytochrome c release was observed (Fig. 7b). All doses of RAgNPs treated cells showed significant (*p* < 0.001) release of cytochrome c compared to control cells; the fold increase observed were 4.11, 5.5 and 6.4 for 2.5, 5 and 10 µg/mL, respectively.

**Figure 8:**
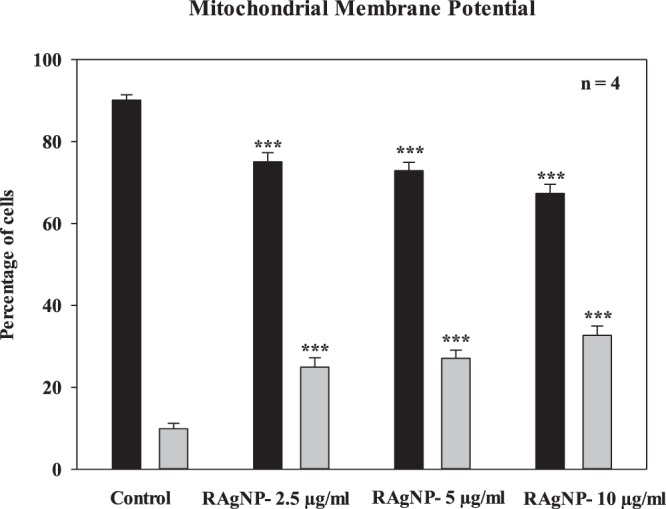
Evaluation of mitochondrial membrane potential using the flow cytometric analysis of JC-1 fluorometric stain. Percentage of polarized (black) and depolarized (grey) mitochondrial membrane potential were determined and compared to the percentage of the corresponding mitochondrial membrane potential of untreated cells. Only the PDT-treated cells showed a change in mitochondrial membrane potential (***p < 0.001).

To further confirm that RAgNPs induced apoptosis, mitochondrial membrane potential or mitochondrial destabilization was measured in MCF-7 cells after the treatment with RAgNPs. Figure 8 shows that RAgNPs induced significant damage to mitochondria after 24 h incubation. The percentages of polarized and depolarized membrane potential in each group was determined and compared to the respective percentage of the control cells. After 24 h of incubation with JC-1 stain, no change in membrane potential was detected in control cells. However, changes in both polarized and depolarized cell populations were noticed in RAgNPs treated cells. The treated cells increased the depolarized mitochondrial membrane and decreased the polarized membranes. All the tested concentrations of RAgNPs (2.5, 5 and 10 μg/mL) significantly (*p* < 0.001) increased the depolarized mitochondrial membrane (24.95 ± 2.25, 27.08 ± 2.01, 32.67 ± 2.27%) and decreased the polarized mitochondrial membrane (75.05 ± 2.25, 72.93 ± 2.01 and 67.33 ± 2.27%).

In order to recognize the cell death pathway induced by RAgNPs, we examined the activation of two specific effector caspases (caspases 3 and 7), release of cytochrome c and the changes in mitochondrial membrane potential. The Annexin V/PI staining showed the induction apoptosis by the increased percentages of apoptotic cells in the treated groups. The increased cytotoxicity and decreased proliferation in cells is due to various factors such as size of the NPs, Ag content and capping agents. Small NPs exhibited higher toxicity due to the enhanced cellular uptake and large surface area for interaction with biomolecule^35,36^. Apoptosis and necrosis, the most preferred cell death mechanisms^37^ investigated upon AgNPs exposure. Annexin-V FITC was used as an apoptotic marker while PI used to detect membrane integrity to identify necrosis^38^. Our results clearly showed the increased early and late apoptotic cells percentage upon treatment, which encourages us to trace the apoptotic signalling pathway. Apoptosis is related with the ROS generation and JNK activation^39^, mitochondrial depolarisation^40^, caspase upregulation^41^, and calcium overload^42^ or by death-inducing signals^43^.

Ag NPs perform well as cancer therapeutics because they can disrupt the mitochondrial respiratory chain, which induces the ROS generation, DNA damage and ATP synthesis^18^. Upon treatment with AgNPs or cisplatin, MCF-7 cells showed decreased Bcl-2 expression and increased Bax expression, representing the mitochondrial connection in cell death^44^. Mitochondria function as critical centres of signalling; various apoptotic regulators can compromise the mitochondrial integrity. The ROS generation by AgNPs may also require mitochondrial involvement, which initiates the intrinsic apoptotic pathway^45,46^. Our results are in agreement with this statement; the treatment of MCF-7 cells with Ag NPs induced cytochrome c release and eventually changed the mitochondrial membrane potential. Mitochondria are known to be involved in programmed cell death; we investigated the changes in mitochondrial membrane potential in terms of JC-1 fluorochrome. In our study, the strong dissipation in mitochondrial membrane potential in the RAgNPs exposed MCF-7 cell line suggests a possible dysfunction of cellular mitochondrial membrane after treatment. The loss of mitochondrial membrane potential might be the reason for the involvement of apoptosis in the AgNPs treated cells^47^. Small changes in particle size and functional groups on the surface of NPs affect the cell death mechanisms. Additionally, these factors may also have significant effect on NPs and membrane interaction, NP internalization and degradation within cells.

**Figure 9:**
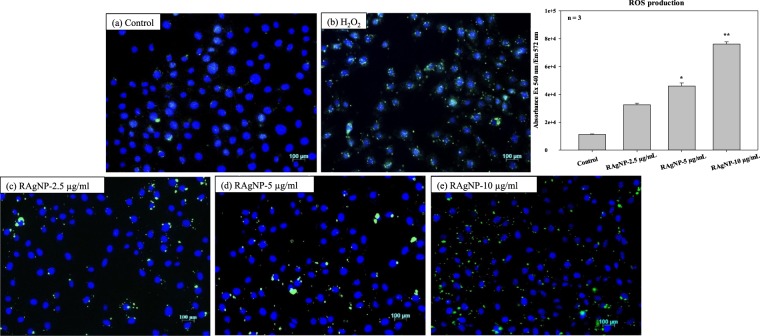
Effect of RAgNPs on ROS production; the green signals showing increased production of ROS upon treatment, in the control (**a**) slide ROS production is less compared with the RAgNPs treated groups (**c**–**e**); H_2_O_2_ treated group served as the positive control. The quantitative measurement of the ROS (**b**) showed that there is significant increase in ROS in RAgNPs treated groups in a dose dependent manner.

Basically, Ag NPs cytotoxicity occurred due to their chemical transformation of neutral silver (Ag°) to Ag^+^, Ag-O-, Ag-S-, which lead to ROS production by chain effect (Fig. 4). The dynamically chemical and biological changes were observed on ROS production. The generation of ROS was evaluated after exposure to various concentrations of RAgNPs. An increase in the intracellular ROS levels was observed, as shown in Fig. 9. H_2_O_2_ treated cells were kept as positive control. The increased ROS production observed in the H_2_O_2_, 2.5, 5 and 10 µg/mL of RAgNPs treated groups compared with the control. These results reflects that RAgNPs are capable of inducing cytosolic oxidative stress and thereby promote cell death. Similarly, the groups treated with RAgNPs (2.5, 5 and 10 µg/mL) showed a significant nuclear damage in Hoechst satin. The Hoechst nuclear stain was used to measure the level of DNA damage. The control cells showed dense spherical homogenously stained nuclei, whereas the cells treated with RAgNPs showed irregular nuclear shape, scattered nuclear granules suggestive of nuclear fragmentation (Fig. S2).

The accumulation of ROS disturb the redox control of cell cycle progression via phosphorylation and ubiquitination of cell cycle regulatory proteins such as cyclins, Cdks and Cdk inhibitors, leading to aberrant cell proliferation and apoptosis^48^. Our results revealed that the release of cytochrome c and caspase 3/7 activity were up regulated in a ROS-dependent manner in MCF-7 cells, the nuclear degeneration observed by Hoechst stain also supports the results (Fig. S2). The toxicity of RAgNPs on WS1 fibroblast cells (normal) were tested and the results are shown in Fig. S3. The RAgNPs are not cytotoxic to the normal cells compared to the cancer cells. The morphological evaluation of WS1 cells after the 24 h treatment did not show more dead cells, which also no signs of cytotoxicity in LDH assay and did not influence on the proliferation in ATP assay, which indicating the less toxicity. Mitochondrial and cellular reactive oxygen species (ROS) play important roles in both physiological and pathological processes. Different ROS, such as superoxide, hydrogen peroxide, and peroxynitrite, stimulate distinct cell-signalling pathways and lead to diverse outcomes depending on their amount and subcellular localization^49^.

**Figure 10:**
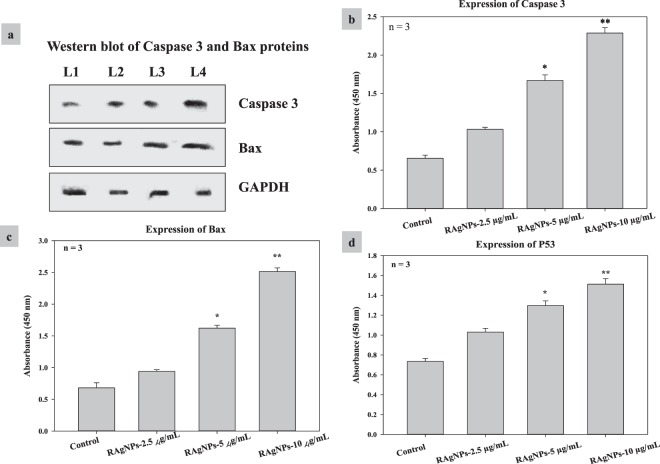
Effect of RAgNPs on apoptotic protein expression of caspase 3, Bax by western blotting (**a**) (L1-Control; L2- RAgNPs-2.5 µg/mL; L3- RAgNPs-5 µg/mL; L4- RAgNPs- 10 µg/mL). (**b**–**d**) shows the effect of RAgNPs on caspase 3, Bax and P53 expression by ELISA. The results showed that the proapoptotic proteins such as P53, caspase 3 and Bax levels were significantly increased in RAgNPs treated groups than control cells.

To investigate the effect of RAgNPs in inducing the intrinsic apoptotic pathway we examined the expression of caspase 3, Bax by western blotting (Fig. 10a) and caspase 3, Bax and P53 expression by ELISA (Fig. 10b–d). In cancer cells the proapoptotic proteins level will be low compared with apoptotic cells. In our experiments, the proapoptotic proteins such as P53, caspase 3 and Bax levels were high in treated groups than control cells. The RAgNPs at 5 and 10 µg/mL concentrations showed significant (*p* < 0.05 and *p* < 0.01) expression of all three proteins tested. Upon treatment active P53, caspase 3 and Bax highly expressed in nucleus. The activation of these proteins leads to mitochondrial permeability; these results were supported by the mitochondrial membrane potential assay and cytochrome c release.

Caspase 3, play a central role in the execution of apoptosis by activating the cleavage of PARP. The sequence at which caspase 3 cleaves PARP is very well conserved in the PARP protein from very distant species, indicating the potential importance of PARP cleavage in apoptosis^50,51^. In the present study, we have examined the expression of caspase 3 and subsequent apoptosis in MCF-7 cells treated with RAgNPs. PARP is cleaved by caspase-3 early during apoptosis in many different cell lines. P53 is a multifunctional tumor suppressor that regulates DNA repair, cell cycle arrest, apoptosis, cell survival as well as oxidative stress. P53 responds to a wide range of cell death stimuli, which can induce apoptosis by activating gene expression, or by permeabilizing mitochondria. P53 accumulates in the nucleus and control the expression of proapoptotic member, Bax^52^. P53 can translocate to mitochondria, interact with the antiapoptotic Bcl-2 proteins, neutralise them and cause apoptosis^53^. The present study showed the expression of P53 in MCF-7 cells when treated with RAgNPs. P53 directly activate the proapoptotic Bcl-2 protein Bax to permeabilize mitochondria and engage in apoptosis. The Bcl-2 family proapoptotic proteins, Bax and Bak, are major regulators and effectors of mitochondrial outer membrane permeabilization (MOMP). Whereas the antiapoptotic members suppress MOMP by inhibiting the activation of Bax/Bak. The proapoptotic proteins, considered the stress signals, promote MOMP by direct or indirect activation of Bax/Bak. Once activated, both Bax and Bak form homo-oligomers on the outer mitochondrial membrane, and generate membrane pores, which further allow the release of apoptogenic proteins cytochrome c from the intermembrane space, leading to the formation of apoptosome and subsequent effector caspase activation thereby the execution of mitochondrial intrinsic apoptosis^54,55^. Our results showed the significant release of cytochrome c and mitochondrial membrane permeabilization, which is directly, linked with the expression of the proapoptotic proteins Bax and the expression of caspase 3 and P53 apoptotic mediators in western blotting and ELISA experiments. Therefore, the induction of P53 regulated apoptosis in MFC-7 breast cancer cells upon RAgNPs treatment is a major target for cancer therapy.

These findings suggest that RAgNPs significantly induced mitochondria mediated caspase dependant apoptosis. The molecular mechanism of RAgNPs mediated up-regulation of caspases should be investigated further.

## Conclusion

We demonstrated biological green synthesis of AgNPs using *Rubus* extract. The successful formation of *Rubus* extract layers on the surface of Ag NPs was confirmed by SEM, TEM, FTIR, and XRD measurements. The synthesised RAgNPs showed antiproliferative effects on MCF-7 breast cancer cells in a dose dependent manner. Interestingly, it was observed that anticancer activity of RAgNPs is strongly associated with the induction of apoptosis by mitochondrial pathway. Apoptosis was significantly higher in the RAgNPs treated cells with increased caspase 3/7 activity, cytochrome c release (*p* < 0.001) and increased percentages of early and late apoptotic cells (*p* < 0.001). All doses of RAgNPs (2.5, 5 and 10 μg/mL) significantly (*p* < 0.001) increased the depolarized mitochondrial membrane (24.95, 27.08 and 32.67%) and decreased the polarized mitochondrial membrane (75.05, 72.93 and 67.33%) due to its cytotoxic behaviour. Our results showed significant increases in cytotoxicity, caspase 3/7 activity, cytochrome c release, depolarized mitochondrial membrane potential, nuclear damage, ROS production and a decrease in cellular proliferation, which ultimately support the high uptake and anticancer activity of RAgNPs. The highest concentrations of RAgNPs showed significant (*p* < 0.05 and *p* < 0.01) expression of caspase 3, Bax and P53 proteins in ELISA and western blotting experiments. We have shown mechanistically AgNPs activates the intrinsic apoptotic pathway in MCF-7 breast cancer cells. Further studies will be conducted to determine the cytotoxic potential of other medically sound extracts and available cancer drugs with Ag NPs on cancer cells.

## Methods

### Materials

All required chemicals were procured from Sigma-Aldrich, South Africa and used directly without any additional purification.

### Preparation of *Rubus fairholmianus* root acetone extracts (RFRA)

*R*. *fairholmianus* was collected from India and authenticated (voucher specimen no: BSI/SRC/ 5/23/2010-11/Tech.1657) by Botanical Survey of India. The root, stem and leaves were extracted in Soxhlet using acetone and the dried extract dissolved in 0.5% DMSO for further analysis. The preliminary screening showed that root possessed high content of phytochemicals and higher radical scavenging property.

### Synthesis of *Rubus* extract conjugated Ag nanoparticles

The *Rubus* extract conjugated Ag NPs (RAgNPs) were synthesized by *R*. *fairholmianus* root acetone extracts (RFRA) mediated bio-reduction of silver nitrate. Briefly, 15 mM of silver nitrate salt (AgNO_3_, purity >99%, purchased from Sigma-Aldrich, South Africa) was dissolved in 200 mL of deionized water using magnetic stirring at room temperature. Then, 0.6 g of prepared RFRA was introduced into the solution. The RFRA extract capped the silver ions and reduced them into Ag NPs within 5–6 min. The fast colour change of reaction solution from yellow to brown exhibited the formation of Ag NPs. Further, the reaction mixture was left stirring for 6 h at 30 °C. After completion, reaction mixture was centrifuged (7000 rpm) multiple times using deionized water and ethanol to remove excess extracts.

### Materials Characterizations

The ultraviolet-visible (UV-Vis) spectrum of as-prepared sample was carried out using Shimadzu UV-2450. Transmission Electron Microscope (TEM, 200 kV, JEOL-JEM-2100, Japan) was used to characterise internal morphologies of RAgNPs. A diluted suspension in ethanol was prepared and the cupper grid was twice dipped in this suspension. Sample grid was properly dried before measurement. Scanning Electron Microscopy (SEM) images were received using TESCAN, VEGA SEM under a 20 kV electron acceleration voltage coupled with an energy dispersive X-ray spectrum (EDS). The powder XRD pattern was recorded using Philips PAN Analytical X’Pert X-ray diffractometer equipped with a Ni-filtered Cu Kα radiation source of the 0.15418 nm. The instrument was operated at 40 kV and 45 mA with a scanning rate of 1°/min for scan angles of 5 to 90°. The photoluminescence spectrum at room temperature was acquired using Perkin Elmer (LS 45 Fluorescence Spectrometer, 230 V). Attenuated Total Reflectance-Fourier Transform Infrared Spectroscopy (ATR-FTIR) measurement was carried out using Perkin Elmer Spectrum 100 FTIR spectrophotometer.

### Cell culture

Human breast cancer cells MCF-7 (ATCC HTB-22) were used for the *in vitro* studies. The cells were cultured in Dulbecco’s Modified Eagle Medium (DMEM) media with 10% heat inactivated Fetal Bovine Serum (FBS) (FBS; Gibco 306.00301). The medium was supplemented with1% penicillin/streptomycin (PAA Laboratories GmbH, P11-010) and 1 µg/mL Amphotericin B (PAA Laboratories GmbH, P11-001).

Human skin fibroblast monolayer cultures (WS1-ATCC CRL1502) were grown in Eagle’s minimal essential medium (Invitrogen 32360–026) that was modified to contain 2 mM L-glutamine (Gibco, 25030), 1 mM sodium pyruvate (Gibco, 11360), 0.1 mM nonessential amino acids (Gibco, 11140), 1% amphotericin-B (Gibco, 104813), 1% penicillin–streptomycin (Gibco, 15140) and 10% v/v foetal bovine serum (FBS; Gibco, 306.00301).

Cells were maintained in a CO_2_ incubator (37 °C, 5% CO_2_ and 80% humidity). Cells were washed with Hank’s Balanced Salt Solution (HBSS, Invitrogen, 10–543 F) when it became confluent and detached with TryplExpress (Gibco, 12604) and subcultured. Cells were seeded at a concentration of 5 × 10^5^ cells/plate in 3.5 cm^2^ diameter culture plate.

### Morphological evaluation

Analysis of cellular morphological changes in cells after the treatments with RAgNPs was important for the preliminary evaluation of cytotoxic effects. After 24 h of incubation with different concentrations of RAgNPs (2.5, 5 and10 µg/mL), the cells were rinsed with HBSS and replenished with fresh medium, then the morphological changes were noticed using Wirsam, Olympus CKX 41 inverted light microscope.

### Proliferation and cytotoxicity

CellTiter-Glo^1^ luminescent assay (Promega, G7571, Anatech Analytical Technology, South Africa) quantifies the ATP levels in metabolically active cells. Briefly, 50 µl of ATP reagent and cell suspension was incubated at room temperature for 10 min in dark and the luminescence was measured using 1420 Multilabel Counter Victor3 (Perkin-Elmer, Separation Scientific). The Cyto-Tox96 X assay (Anatech, Promega G 400) was used to evaluate the cytotoxic activity of C1 and C2 on MCF-7 cells. The membrane integrity was assessed by quantifying the Lactate Dehydrogenase (LDH) released to the media following the pre-treatment with RAgNPs. The LDH reagent and culture medium (50 µl each) were mixed and incubated in dark at room temperature for 30 min and colorimetric compound was read at 490 nm (Perkin-Elmer, VICTOR3™). The more LDH released to the culture medium the more cytotoxic. The proliferation and cytotoxicity were evaluated based on a comparison with untreated cells and the IC_50_ values were calculated.

### Annexin V/PI double staining

The Annexin V-fluorescein isothiocyanate (FITC) apoptosis detection kit (Becton Dickinson, 556570, Scientific Group, South Africa) was used to distinguish the population of apoptotic and non-apoptotic cells. The cell suspension (1 × 10^6^/mL) (100 µl) was stained with 5 µl of Annexin V and propidium iodide (PI), vortexed and incubated for 10 min at room temperature in dark. After the staining, flow cytometry was performed using Fluorescence Activated Cell Sorting (FACS) Aria flow cytometer (BD Bioscience) to quantify the population of apoptotic and non-apoptotic cells.

### Caspase 3/7 activities and cytochrome c release

The downstream executioner enzymes caspase 3/7 activities was evaluated using the Caspase-Glo 3/7 luminescent kit (Promega G8091, Whitehead Scientific, Bracken fell, South Africa) for the determination of caspase activity. Equal volume (50 µl) of RAgNPs pre-treated cells and caspase reagent was seeded in 96-well luminous plate (Scientific Group Adcock Ingram, Midrand, South Africa BD354651), incubated for 3 h at room temperature. The cleavage of substrate by caspases was measured by generated luminescent signal and was measured using Victor^3^ (Perkin-Elmer, Separation Scientific).

An apoptotogenic enzyme, cytochrome c is vital for apoptotic events. An Enzyme-linked immunosorbent assay (ELISA) (human cytochrome c Platinum ELISA kit) was used to quantify the cytochrome c in cytosol. Briefly, the resuspended cells were lysed and the lysate was spun and a 50-fold dilution of the supernatant was done with 1 x assay buffer. Lysate was further diluted (1:2); a volume of 100 μl sample was added to all wells containing 1x assay buffer. Fifty microliters of biotin-conjugated anti-human cytochrome c antibody was added and incubated for 2 h at room temperature. Thereafter, the plate was washed three times with 400 μl wash buffer and Streptavidin-HRP secondary antibody (100 μl) was added and incubated for 1 h. Tetramethyl-benzidine (TMB) (100 μl) substrate was added after washing and incubated for 10 min. Finally, the reaction was stopped and absorbance was measured at 450 nm using the Victor^3^ (Perkin-Elmer, Separation Scientific).

### Mitochondrial membrane potential analysis

The evaluation of mitochondrial membrane potential (*ΔΨm*) variations is an indirect measure of intrinsic apoptosis pathway. *ΔΨm* were analysed using BD^TM^ Mito Screen flow cytometry mitochondrial membrane potential detection kit (Cat No. 551302). 1^st^ J-aggregate-forming cationic dye (JC-1), is a fluorochrome used to evaluate the *ΔΨm*. Viable cells with polarized *ΔΨm* able to take up the JC-1 stain to form mitochondrial JC-aggregates with a red spectral shift and fluorescence. Damaged cells with depolarized mitochondria cannot take up JC-1 stains, do not fluoresce and JC-1 remains in the cytoplasm as its monomer. Briefly, the cells were resuspended in HBSS (1 mL) and spun at 1200 rpm for 5 min at 4 °C. The cells were dissolved in JC-1 working solution (0.5 mL). The mixture was incubated at 37 °C in a CO_2_ incubator for 15 min, followed by washing (twice) with 1 mL of 1 x assay buffer and centrifuged at 1200 rpm. Finally, the cells were dissolved in 0.5 mL of 1 x assay buffer, vortexed and analysed using Fluorescence Activated Cell Sorting (FACS) Aria flow cytometer (BD Biosciences).

### Reactive Oxygen Species (ROS) assay and Hoechst staining

The cellular ROS produced was measured by Dichlorodihydrofluorescein diacetate (DCFH-DA) assay. Briefly, the cells were cultured in 3.4 cm^2^ diameter culture dishes over sterile cover slips for 20 h and were treated with 100 μM DCFH-DA for 30 min in dark. Followed by DCFH-DA reaction, the cells were treated with RAgNPs for 12 h. The nuclear stain, 4′−6-Diamidino-2-phenylindole (DAPI, Invitrogen, D1306) (1 μg/mL) were added and incubated for 10 min in dark. Following the completion of the DCFH-DA and DAPI reaction, the cells were washed thrice with PBS (2 mL). The cover slips were fixed on clean glass slides using mounting media (Propyl Gallate, Fluka, Sigma-Aldrich, 02370) and were sealed. The slides were examined using the Carl Zeiss Axio Observer Z1. The following filters were used for fluorescent compounds: 358Ex/461Em for DAPI and 494Ex/518Emfor DCFH-DA. For the quantitative analysis of ROS generation, MCF-7 cells were grown in 96 well tissue-culture plates until 80% confluent and treated with various concentrations of RAgNPs. Reactive oxygen species present inside cells were measured using dichlorofluorescein (DCF) fluorescence. 1 µM Dichlorodihydrofluorescein diacetate (DCFH-DA) was added MCf-7 and incubated for 30 min. The fluorescence of oxidized DCFH-DA was read at Ex540 nm/Em572 nm after 1 h using a Victor^3^ (Perkin-Elmer, Separation Scientific).

The nuclear damage induced by RAgNPs was observed by Hoechst staining. Cells were cultured in 3.4 cm^2^ diameter culture dishes over sterile cover slips for 20 h in a culture dish and treated with various concentrations of RAgNPs. After 24 h incubation, cells were stained with 1 μg/mL Hoechst stain (Hoechst 33258, H21491) for 15 min. Thereafter, the cells were rinsed with PBS and the blue fluorescent signal was examined using the Carl Zeiss Axio Observer Z1 with the filter set of 352Ex/461Em wavelength.

### Western blot analysis

The concentration of proteins in cell lysates was assessed using BCA Protein Assay Reagent (Thermo Fisher Scientific, Rockford, IL, USA). Depending on protein concentration, cell lysates were diluted in RIPA buffer to the gel-loading concentration of proteins (2.5 μg/μl), mixed with equal volume of sample buffer (0.125 M Tris/HCl pH 6.8, 10% glycerol, 4% SDS, 0.25 M DTT) and heated for 5–7 min at 110 °C. Protein samples were separated using a protein electrophoresis (Bio-Rad, Hercules, CA). The proteins separated by SDS-PAGE were transferred to PVDF membrane Immun-Blot (Biorad Cat:162–0177) for 3 h at 0.25 A, using a Semi Dry blotter (Sigma-B2529). The membrane was blocked with 5% BSA in TBS for 15–20 min and incubated with respective primary antibody (Caspase 3- mouse monoclonal antibody, Cat# SC-7272, Santa Cruz biotechnology; Bax- mouse monoclonal antibody, Cat# 336400, Life technologies; GAPDH- mouse monoclonal antibody, Cat# MA5-15738, Invitrogen) at 4 °C overnight. After the incubation, the membrane was washed three times (5–10 min) with TBS containing 0.1% Tween-20 and incubated for 2 h with the horseradish peroxidase conjugated secondary antibody (Goat anti-mouse HRP, Cat# SC-2005, Santa Cruz Biotechnology). Afterwards, the membrane was washed with TBS and the chromogenic signal was detected by adding the colour-developing reagent (1% DAB 250 µl + 0.3% H_2_O_2_ 250 µl added to 5 mL of PBS), kept in dark for 5 min, and developed bands were scanned.

### Enzyme Linked Immunosorbent Assay (ELISA)

We used cell based ELISA, to analyse the expression of caspase 3, Bax and P53 in cultured cells. After 24 h treatment, cells were detached using TrypLE^TM^ (Gibco, 12563-029, Life Technologies) and 3 × 10^4^ cells were seeded into a 96-well flat-bottomed microplate (Corning®, Costar® 3596). Cells were allowed to adhere in a total volume of 100 μL media and were fixed using 8% paraformaldehyde (100 μL). After 15 min incubation at room temperature, the plates were washed thrice with PBS and were incubated with 200 μL of 1% permeabilization solution (Triton X-100 in PBS) for 30 min at room temperature. Afterwards Triton X-100 was replaced with 200 μL of 2% blocking solution (10% BSA in PBS; KPL, 50-61-01, Whitehead Scientific) and incubated for 2 h at room temperature. Plates were washed and incubated with 100 μL of 1:30 diluted primary antibody (P53, Cat# SC-99, Santa Cruz biotechnology; Caspase 3, Cat# SC-7272, Santa Cruz biotechnology; Bax, Cat# 336400, Life technologies), for 2 h at room temperature. Plates were washed thrice with 1% wash buffer (400X Tween-20 in PBS) and 100 μL of a 1:5000 secondary antibody was added (Goat anti-mouse IgG-HRP: Cat# SC-2005, Santa Cruz Biotechnology) and incubated for 2 h at room temperature. Followed by rinsing, 100 μL TMB was added and the reaction was stopped by adding 1 M hydrochloric acid and the colorimetric reaction was measured at 450 nm (Perkin-Elmer, Victor^3^ plate reader).

### Statistical analysis

All data were presented as mean ± standard error of the mean (SEM) of at least three tests done in duplicates. The statistical significance was analysed using Sigma Plot version 13.0. The treated groups were compared with the control groups by one way ANOVA to analyse the statistical significance. The *p* value less than 0.05 was measured as significant.

## Electronic supplementary material

supplementary material

## Data Availability

The datasets generated during and/or analysed during the current study are available from the corresponding author on reasonable request.
