# Supplementary material for: Apoptotic efficacy of multifaceted biosynthesized silver nanoparticles on human adenocarcinoma cells
Source: Sci Rep. 2018 Sep 25;8:14368. doi: 10.1038/s41598-018-32480-5 (PMC6156419; doi:10.1038/s41598-018-32480-5)

Apoptotic efficacy of multifaceted biosynthesized silver nanoparticles on human adenocarcinoma cells

Blassan Plackal Adimuriyil George[a*], *Neeraj Kumar*[b,c], *Heidi Abrahamse*[a], *Suprakas Sinha Ray*[b, c]
[a]*Laser Research Centre, Faculty of Health Sciences, University of Johannesburg, Doornfontein 2028, South Africa*
[b]*Department of Applied Chemistry, University of Johannesburg, Doornfontein 2028, South Africa*
[c]*DST/CSIR National Centre for Nanostructured Materials, Council for Scientific and Industrial Research, Pretoria 0001, South Africa*

Corresponding Author: Blassan P. George
Address:
Laser Research Centre,
Faculty of Health Sciences,
University of Johannesburg,
P.O. Box 17011,
Doornfontein 2028, South Africa
Telephone:+27 11 559 6926
Fax:+27 11 559 6558
E-mail address:blassang@uj.ac.za; blassanpgeorge@gmail.com

Figure S1. Zeta potential distribution of RAgNPs

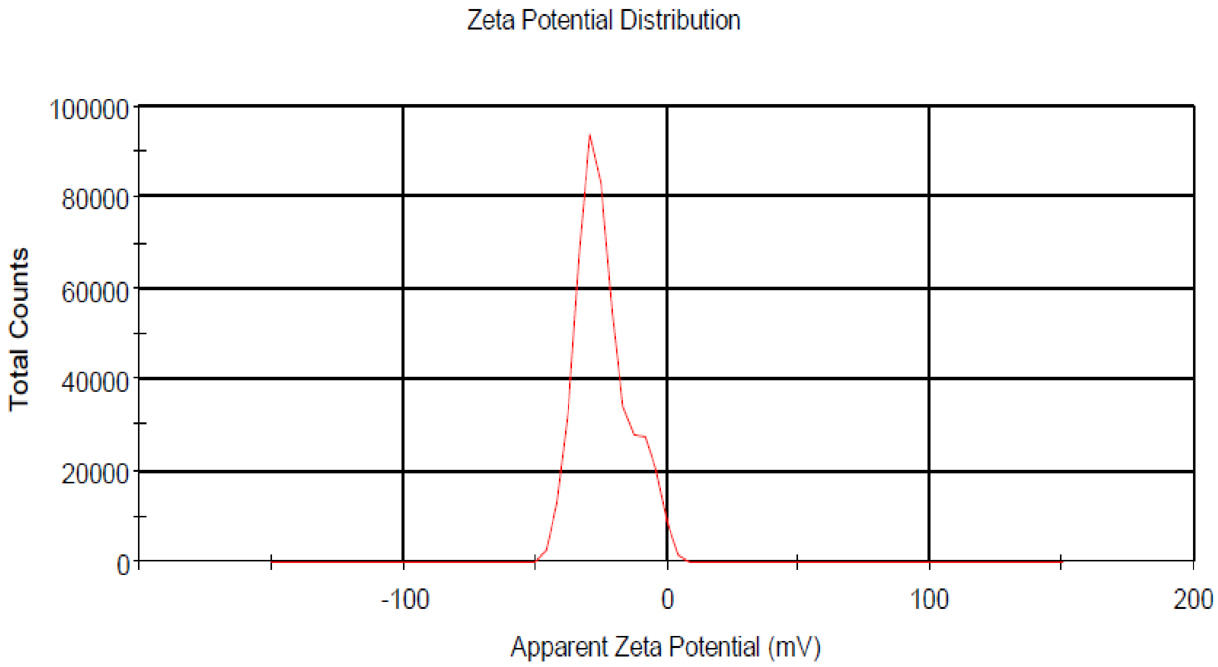

Figure S2. Hoechst staining

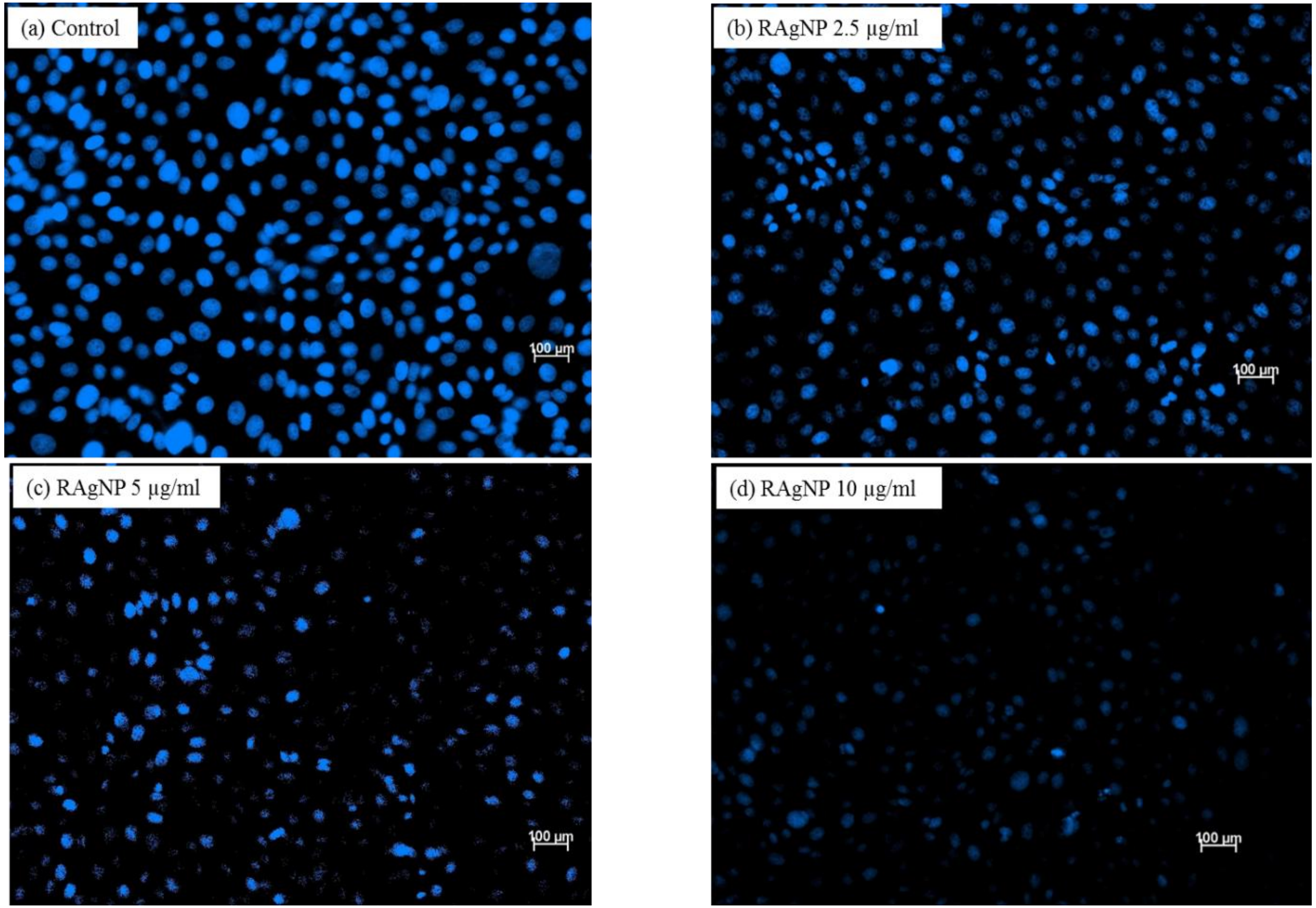

Figure S3
Morphological changes in WS1 cells

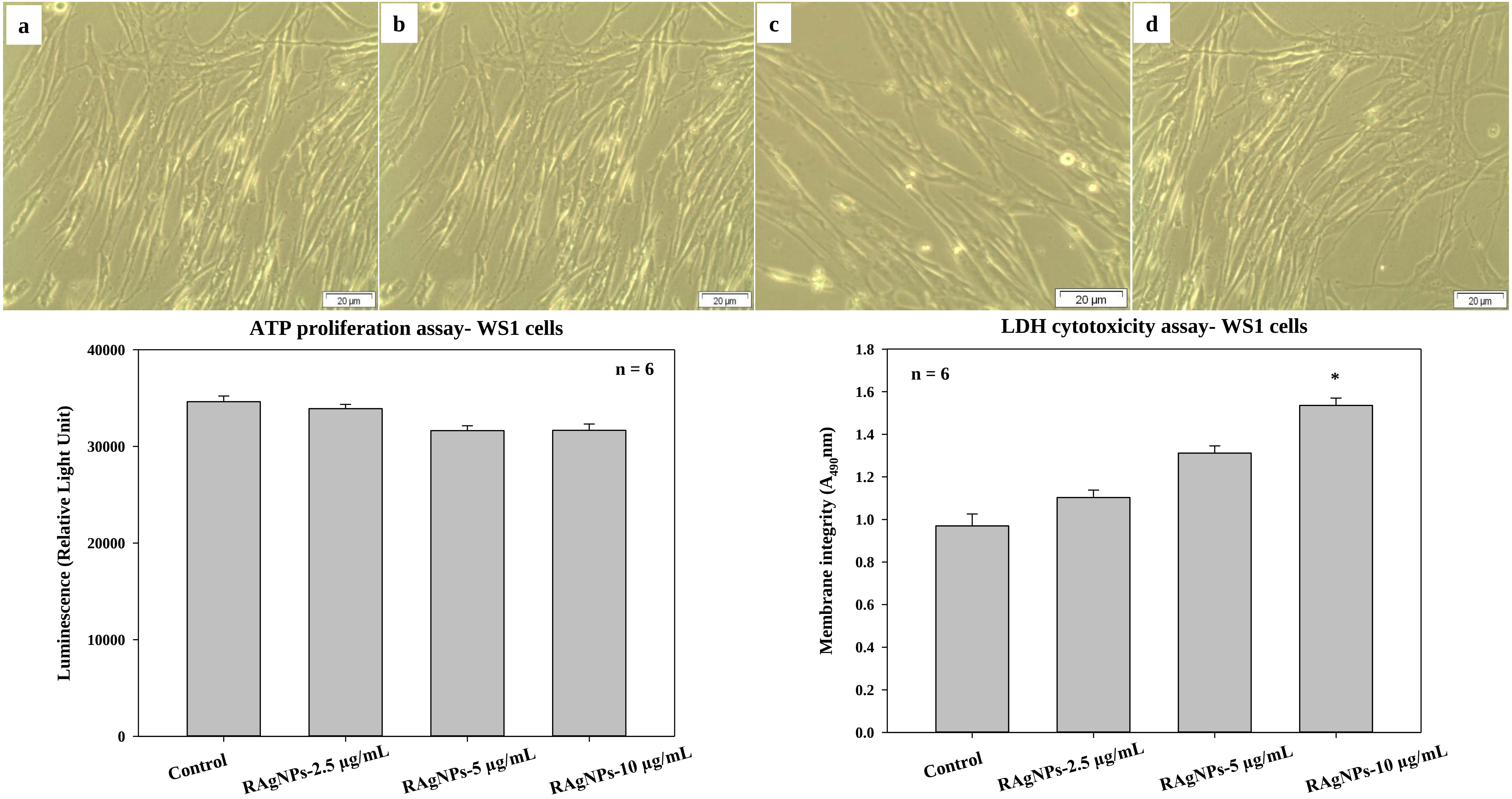

Supplement: Supplementary file 1 — supplementary material [file 41598_2018_32480_MOESM1_ESM.pdf]
